# Behavioral and transcriptomic effects of the cancer treatment tamoxifen in mice

**DOI:** 10.3389/fnins.2023.1068334

**Published:** 2023-02-10

**Authors:** Elena Galvano, Harshul Pandit, Jordy Sepulveda, Christi Anne S. Ng, Melanie K. Becher, Jeanne S. Mandelblatt, Kathleen Van Dyk, G. William Rebeck

**Affiliations:** ^1^Department of Neuroscience, Georgetown University Medical Center, Washington, DC, United States; ^2^Department of Pharmacology and Physiology, Georgetown University Medical Center, Washington, DC, United States; ^3^Department of Oncology, Georgetown Lombardi Comprehensive Cancer Center, Georgetown University, Washington, DC, United States; ^4^Department of Psychiatry, UCLA Semel Institute for Neuroscience and Human Behavior, Los Angeles, CA, United States

**Keywords:** tamoxifen, chemotherapy, blood-brain barrier, transcriptomic (RNA-seq), behavior, mouse model, chemotherapy-induced cognitive impairment

## Abstract

**Introduction:**

Tamoxifen is a common treatment for estrogen receptor-positive breast cancer. While tamoxifen treatment is generally accepted as safe, there are concerns about adverse effects on cognition.

**Methods:**

We used a mouse model of chronic tamoxifen exposure to examine the effects of tamoxifen on the brain. Female C57/BL6 mice were exposed to tamoxifen or vehicle control for six weeks; brains of 15 mice were analyzed for tamoxifen levels and transcriptomic changes, and an additional 32 mice were analyzed through a battery of behavioral tests.

**Results:**

Tamoxifen and its metabolite 4-OH-tamoxifen were found at higher levels in the brain than in the plasma, demonstrating the facile entry of tamoxifen into the CNS. Behaviorally, tamoxifen-exposed mice showed no impairment in assays related to general health, exploration, motor function, sensorimotor gating, and spatial learning. Tamoxifen-treated mice showed a significantly increased freezing response in a fear conditioning paradigm, but no effects on anxiety measures in the absence of stressors. RNA sequencing analysis of whole hippocampi showed tamoxifen-induced reductions in gene pathways related to microtubule function, synapse regulation, and neurogenesis.

**Discussion:**

These findings of the effects of tamoxifen exposure on fear conditioning and on gene expression related to neuronal connectivity suggest that there may be CNS side effects of this common breast cancer treatment.

## 1. Introduction

Breast cancer is the most common female cancer worldwide, and the second largest cause of cancer death in women (Lei et al., 2021). Of the different subtypes, estrogen receptor-positive breast cancer (ER+) is the most frequent, accounting for about 70% of all diagnosed breast cancer cases (Chen, 2011). ER + breast cancer tumors overexpress ERs and their proliferation is driven by the interaction of these receptors with estrogen. Since these tumors rely on estrogen binding for their growth, endocrine therapies that prevent this interaction and the subsequent activation of estrogen-dependent pathways have shown success in reducing the growth of breast cancer cells. These therapies have been the standard adjuvant treatment for ER + tumors since the 1970’s (Almeida et al., 2020). The most common endocrine therapy agents are tamoxifen, a selective estrogen receptor modulator that competitively binds to ERs, and aromatase inhibitors, which reduce endogenous estrogen synthesis (Lumachi, 2013). Tamoxifen is one of the most frequently prescribed drugs in the treatment of ER + breast cancer among younger women because it can be prescribed at any age or menopausal status, while aromatase inhibitors are recommended for use with post-menopausal women (Palmer et al., 2008). With treatment duration recommendations of 5–10 years (Burstein et al., 2019), the potential exposure to TAM among younger women with ER + breast cancer is extensive.

As a selective estrogen receptor modulator, tamoxifen could influence the activity of target estrogen sites beyond tumor cells, including the brain, since it crosses the blood-brain barrier. The antagonistic action of tamoxifen on ERα (Mandlekar and Kong, 2001) may lead to an abrupt drop in estrogen-related activity in the brain. Estrogen has long been known to play a critical role in women’s brain health, having both genomic effects (e.g., regulating neuronal function) and non-genomic effects (e.g., contributing to rapid neuronal firing *via* membrane-bound receptors). Treatment with tamoxifen could therefore interfere with estrogen-regulated cognitive functions, especially in pre-menopausal women, who have high circulating estrogen levels prior to tamoxifen therapy (Palmer et al., 2008). Clinical studies, such as clinical trials and prospective cohort studies, have resulted in somewhat mixed evidence about the effects of tamoxifen on cognition (Schilder et al., 2010; Boele et al., 2015; [Bibr B49][Bibr B67]). Examining the cognitive effects of tamoxifen in humans is complicated by many factors though, such as other concomitant cancer treatments and an inability to tightly control drug exposure.

A well-controlled and accessible approach toward a better understanding of the effects of endocrine therapies on the brain and cognition is studying their effects in animal models. Pre-clinical animal models have been used in the study of the cognitive effects of other cancer treatments, such as doxorubicin and methotrexate (Winocur et al., 2018; Matsos and Johnston, 2019). These studies allow the investigation of brain pathophysiological mechanisms, avoiding the variability of drug exposure, genetics, and cancer treatment regimens in humans (Staay et al., 2009; Webster et al., 2014). In this study, we employed a mouse model to explore the effects of long-term administration of tamoxifen on select behavioral assays informed by clinical evidence and clinical experience with this population, as well as CNS gene expression. These studies were complemented by mass spectrometry analysis of tamoxifen in the brain and by analysis of mRNA species in the brain altered by tamoxifen exposure. Our goals are to address the important question of whether there are potential adverse effects of tamoxifen on brain function among women being treated for breast cancer (Lien et al., 1991; Overk et al., 2012; Zwart et al., 2015) and explore areas in need of future research. As the field continuously evolves, including treating women at risk for cancer with TAM as chemoprevention (Ball et al., 2019), this and future work could help inform treatment development and recommendation guidelines.

## 2. Materials and methods

### 2.1. Ethics statement

This study was conducted in accordance with ethical standards and relevant national and international guidelines for animal welfare, including the National Institutes of Health Guide for the Care and Use of Laboratory Animals. All procedures and handling of the animals were performed according to protocols approved by the Georgetown University Medical Center Institutional Animal Care and Use Committee.

### 2.2. Animal population

**FIGURE 1:**
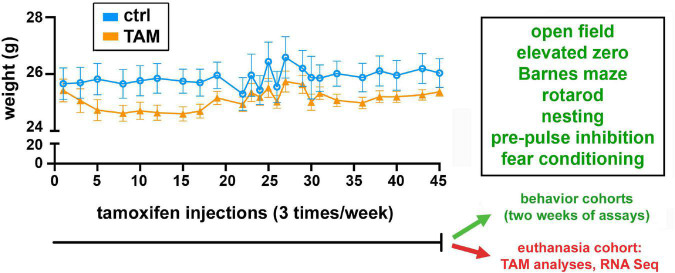
Timeline of tamoxifen or vehicle exposure and associated weight changes, behavioral assessments, and euthanasia. All cohorts of wild-type mice were treated with tamoxifen (TAM) or vehicle control (CTRL) for 45 days. Mice were injected three times per week, and weighed each time. One cohort of 15 mice was then euthanized for tamoxifen metabolite and RNA sequencing analyses. Two other cohorts of 16 mice each were used in the series of indicated behavioral tests over 2 weeks.

Female C57BL/6J mice purchased from Jackson Laboratories at 25 weeks of age were used in this study; at this age, mice are pre-menopausal. Mice were housed four per cage on a 12-h light-dark cycle. Mice had free access to water and rodent chow throughout the study. Treatment was initiated approximately 2 weeks after the mice had arrived, and body masses were measured before each injection. Each mouse was randomly assigned to either the treatment or control groups, and research staff were blinded to treatment status for the metabolite, behavioral, and RNA assays. One cohort of 15 mice (7 controls, 8 TAM treated) were used only for collection of brain tissues for the metabolite analyses (*n* = 6) and RNA sequencing analysis (*n* = 5); two other cohorts (16 per treatment group; a total of 32 mice) were used for behavioral assays (Figure 1). Thus, the metabolite and transcriptomic analyses were unaffected by behavioral assays.

### 2.3. Tamoxifen administration

Tamoxifen (Sigma-Aldrich, T5648) was dissolved in ethanol and diluted in corn oil to obtain a final concentration of 1 mg/ml and 5% ethanol. Accordingly, the vehicle control was prepared as corn oil containing 5% ethanol. Subcutaneous injections of tamoxifen were performed 3 times per week at a dose of 2 mg/kg or an equal volume of vehicle control for 45 days. At that time, mice were either euthanized for tissue collection (cohort 1), or enter the 2 weeks of behavioral assessments (cohorts 2 and 3).

### 2.4. Behavioral assessments

Mice were behaviorally tested using a series of assessments during the daytime of the light/dark cycle according to the schedule shown in Figure 1, for 2 weeks after tamoxifen treatments. As a precaution against stress effects, individuals responsible for mouse injections were not involved in behavioral testing. Prior to each procedure, the mice were habituated to the testing room for 30 min in their home cage. Each apparatus was thoroughly cleaned and wiped down with 70% ethanol prior to each test and between trials to eliminate the potential influence of olfactory cues.

#### 2.4.1. Open field task

The Open Field apparatus consists of a plain plexiglass field of 43 cm^2^. The outermost 8 cm of the field was considered the “outer zone;” the remaining region is the “inner zone”. This task was used to assess locomotion, anxiety, and exploratory behavior. Time spent in the inner zone was used as a measure of anxiety. Total distance traveled was used to evaluate locomotion. Mice were tested one week prior to the initial tamoxifen or vehicle injections and 2 days following completion of treatment.

#### 2.4.2. Elevated zero maze

The Elevated Zero Maze was a ring-shaped platform 600 mm in diameter, with two opposing sections protected by walls 6″ high (the “closed zone”). Mice were initially placed on one of the two zones lacking walls (the “open zone”) and allowed to explore the maze freely for 300 S. Their movement was recorded by an overhead camcorder and analyzed using ANYmaze software (San Diego Instruments). The amount of time spent in the open zone was recorded and used as a measure of exploratory behavior and a negative measure of anxiety. Mice were assessed 1 week prior to the initial tamoxifen or vehicle injections and 2 days following completion of treatment.

#### 2.4.3. Nest-building

Nesting behavior was used to assess general health after treatment. Approximately 1 h before the dark phase the mice were transferred to individual testing cages with no environmental enrichment items. Three grams of pressed cotton squares were weighed and placed in each cage (one square per cage). The mice were individually housed overnight and allowed to shred the cotton square to build their nest. The nests were photographed and assessed the next morning on a rating scale of 1–5 based on the degree to which the square had been torn and built into a nest. The nests were scored in a blinded manner based on comparison to well-defined figures (Deacon, 2006).

#### 2.4.4. Rotarod

The rotarod test was used to assess motor coordination and motor skill learning. The Rotarod apparatus (Model 57624 Ugo Basile Rota-Rod, Stoelting Co.) contains a grooved rod of 30 mm diameter. The mice were placed in adjacent lanes of the apparatus running at the constant speed of four rotations per minute (rpm). When all mice were positioned and facing forward the start button was pressed, and the rod accelerated from 4 to 40 rpm in 300 s. The duration that each mouse stayed on the rotating rod in each trial was recorded as the latency to fall. Latency was automatically recorded, and if a mouse clinging on the rod completed two full passive rotations, the trial was manually stopped for that mouse. The trial ended at 300 s or earlier if all mice had fallen off or were stopped. The standard motor learning task was performed as six trials separated by 15-minute intervals in a single day. During the test, the rod was kept dry and clean with 70% ethanol.

#### 2.4.5. Barnes maze

The Barnes maze was used to assess spatial learning and memory 2 days after the last tamoxifen or vehicle injection. The maze consists of 19 shallow decoy holes and one escape hole located around the edge of a flat table. The maze was in a space containing four extra-maze visual cues, illumination of 150 lx, and 75 dB of white noise. For each trial, the mouse was placed in the middle of the table and allowed to explore the maze freely for 180 s or until it entered the escape hole. If the mouse did not enter the escape hole after 180 s, it was guided to the hole by the experimenter. Mice were habituated to the apparatus one day prior to the start of training. During habituation, the mice were allowed to explore the maze for the duration of one trial (180 s) with the escape hole placed in a location that would not be used for the remainder of the protocol. During each of the four subsequent training days, mice completed four 180 s trials, separated by 15-minute intervals. The training protocol was performed with the escape hole in the target location, which remained constant throughout the remainder of the assessment. After entering the escape hole, the entry hole was covered, and the mouse was allowed to rest for 2 min before being returned to its home cage. Seventy-two hours following the final training trial, each mouse underwent a single probe trial to test for the ability to remember the target hole location. ANYmaze software was used to track and collect data on the animal’s movement during testing.

#### 2.4.6. Pre-pulse inhibition (PPI)

Pre-pulse inhibition was used to assess sensorimotor gating. Testing occurred within sound attenuated startle chambers (SR-Lab Startle Reflex System; San Diego Instruments, San Diego, CA, USA). The 15 min sessions consisted of a 5-min acclimation period with background noise (70 dB), five habituating startling stimuli (105 dB; 40 ms pulse), six blocks of four randomized trials containing pulse-alone (105 dB; 40 ms) and pre-pulse-pulse (pre-pulses: 3, 9, and 12 dB above background noise; 20 ms). During the pre-pulse-pulse trials an inter-stimulus interval of 50 ms (onset to onset) was used. The inter-trial interval ranged from 15–30 s, randomly selected for each trial. Startle amplitude was defined as the peak piezoelectric accelerometer output over a 175-ms period beginning at the onset of the pulse stimulus. Sound pressure levels were calibrated and verified using an SPL meter set to dB(A) weighting and with the microphone positioned at the level of the animals’ ear.

#### 2.4.7. Fear conditioning

Contextual fear conditioning was used to assess amygdala-related learning and memory 2 weeks after treatment was completed. Following habituation, the mice were placed in a novel context, the conditioning chamber for 3 min. The mice then experienced 2 s of 0.75 mA foot shocks each minute for 3 min through the floor grid. After 48 h the mice were re-exposed to the conditioned context, but no shocks followed the 3-minute exploration period. In both the conditioning and the recall trial, 20 decibels of white noise were played as an aversive stimulus to limit jumping behavior. Freezing duration, latency to first freeze, and freezing episodes were quantified by ANYMaze software.

### 2.5. Tissue collection

For metabolite and mRNA analyses, mice from cohort 1 (Figure 1) were euthanized with CO_2_ and intracardiac blood collections were obtained before the animals were intracardially perfused with 0.1 M phosphate-buffered saline solution (PBS, pH 7.4). Brains were cut along the midline, and one hemisphere was fixed while the other hemisphere was dissected into the cerebral cortex, hippocampus, and cerebellum and snap-frozen. Blood collections were spun at 13,000 rpm for 5 min to extract plasma, which was snap-frozen and stored at −80^°^C.

### 2.6. Analysis of tamoxifen and 4-OH-tamoxifen

Ultra-high-performance liquid chromatography-mass spectrometry (UPLC-MS) was employed to measure tamoxifen and its metabolite 4-OH-tamoxifen. Cerebral cortex tissue was cryo-pulverized in liquid nitrogen using mortar and pestle (Coors porcelain mortar and pestle 522-00), and 20–40 mg samples were processed by the addition of 400 μl of methanol containing internal standard and homogenized for 2 min. For plasma samples, 225 μl of methanol containing internal standard was added to 25 μl of plasma. All samples were then incubated on ice for 20 min and incubated at −20°C for 20 min. After centrifugation at 13,000 rpm at 4^°^C for 20 min, the supernatant was analyzed *via* Acquity UPLC BEH C18, 1.7 μm, 2.1 × 100 mm column online with a triple quadrupole mass spectrometer (Xevo-TQ-S, Waters Corporation, USA) operating in the multiple reaction monitoring mode. The sample cone voltage and collision energies were optimized for the analyte to obtain maximum ion intensity for parent and daughter ions using “IntelliStart” feature of MassLynx software (Waters Corporation, USA). Calibration curves for tamoxifen and 4-hydroxytamoxifen (Sigma-Aldrich) were prepared with concentrations ranging from 0.25 pg/ml to 20 ng/ml. Standards were injected at the start and the end of the batch, and calibration curve/quality control (QC) samples were prepared by spiking samples with standard drug. Data were processed using Target Lynx 4.1 and the relative quantification values of analytes were determined by calculating the ratio of peak areas of transitions of samples normalized to the peak area of the internal standard (debrisoquine, Sigma-Aldrich). Water, acetonitrile, isopropanol, and methanol were Optima grade (Fisher Scientific), and high purity formic acid was used (Thermo-Scientific).

### 2.7. RNA extraction and sequencing

A total of 10 mice (*n* = 5 Control, *n* = 5 Tamoxifen) from cohort 1 were used for RNA-sequencing analysis (Figure 1). One hippocampus from each mouse was homogenized in 500 μl of Trizol reagent (Cat # 15596026, Invitrogen) using glass tissue homogenizer. Trizol homogenates were phase separated using chloroform, followed by column-based extraction kit as per manufacturer instruction (Cat # 12183018A, Invitrogen). DNase digestion was performed to remove DNA contaminants from the sample. RNA concentration was measured using nanodrop spectrophotometer (ThermoFisher) and RNA integrity > 9.0 was confirmed *via* TapeStation 2100 bioanalyzer (Agilent Technologies). Further sample QC, library preparations, and sequencing reactions were conducted at GeneWiz (South Plainfield, NJ, USA).

At Genewiz, the RNA sequencing libraries were prepared using the NEBNext Ultra II RNA Library Prep Kit for Illumina using manufacturer’s instructions (New England Biolabs). Briefly, mRNAs were initially enriched with Oligo(dT) beads for cDNA generation. The PCR-enriched sequencing libraries were validated on the TapeStation (Agilent Technologies), and quantified by using Qubit 2.0 Fluorometer (ThermoFisher) as well as by quantitative PCR (KAPA Biosystems). The libraries were multiplexed and clustered onto a flow cell. The flow cell was loaded onto the Illumina HiSeq instrument and the samples were sequenced using a 2 × 150 bp Paired End configuration. Image analysis and base calling were conducted by the HiSeq Control Software. Raw sequence data (.bcl files) generated from Illumina HiSeq was converted into fastq files and de-multiplexed using Illumina bcl2fastq 2.20 software. One mismatch was allowed for index sequence identification.

### 2.8. RNA-sequencing data analysis

All samples were processed in the exact same way and analysis were carried out using R v.4.0.2 suite. After confirming quality of the raw data, sequence reads were trimmed to remove possible adapter sequences and nucleotides with poor quality using Trimmomatic v.0.36. The trimmed reads were mapped to the Mus musculus reference genome GRCm38 using the STAR aligner v.2.5.2b to generate BAM files. Unique gene hit counts were calculated by using featureCounts (Subread v.1.4.6). To identify differentially expressed genes, we used R/Bioconductor DESeq2 v.1.16.1. A Principal Component Analysis (PCA) was performed using the “plotPCA” function within the DESeq2 R package. Hierarchical clustering using Pearson correlation matrix analysis identified one of the tamoxifen-treated samples as outlier, and therefore it was excluded from downstream analyses. Control (*n* = 5) vs. TAM (*n* = 4) group comparison was performed using FDR cut-off = 0.05 and fold-change = 1.5. Genes with adjusted *P*-values < 0.05 were identified as differentially expressed genes. Gene ontology analysis was performed using R/Bioconductor GSEA (pre-ranked fgsea v.1.22). The sequencing data have been deposited in Sequence Read Archive under BioProject accession ID: PRJNA911742.

### 2.9. Statistical analyses

Data are expressed as mean with standard errors of the mean or as mean with standard deviation. Statistical analyses were performed using GraphPad Prism 9.0 software (San Diego) with *p* < 0.05 considered statistically significant. Normality of data was determined by Shapiro-Wilk and D’Agostino-Pearson omnibus normality tests. Data was statistically analyzed by Two-way ANOVA with Šídák’s multiple comparisons test, and multiple unpaired *t*-tests to determine the effects of tamoxifen treatment on animal behavior and cognition.

## 3. Results

### 3.1. Tamoxifen administration

Wild-type C57BL6 mice (27 weeks of age) were treated subcutaneously with tamoxifen in 500 μl corn oil (2 mg/kg) or with vehicle control three times per week over 6 weeks (Figure 1). The pharmacokinetics of tamoxifen required repeated injections due to efficient metabolism by the cytochrome P450 system (Klein et al., 2013). One cohort of tamoxifen-treated and control mice were used for analysis of tamoxifen metabolism in the cerebral cortex (*n* = 6/group) and mRNA in the hippocampus (*n* = 5/group) (Figure 1).

### 3.2. Tamoxifen penetration of the brain

We developed a method of analysis of tamoxifen and 4-OH-tamoxifen to determine brain levels at the end of the exposure period. By UPLC-MS, the quantification range for tamoxifen and 4-OH-tamoxifen were 2.5 pg/ml to 10 ng/ml and 5 pg/ml to 20 ng/ml, respectively. Due to matrix effects, the drug responses varied from 87–100% (tamoxifen) and 85–104% (4-OH-tamoxifen) in plasma and from 104–133% (tamoxifen) and 103–151% (4-OH-tamoxifen) in tissue. Coefficient of variance remained less than 8%, and no sample-to-sample carryover was observed.

We found tamoxifen in the plasma of the treated mice, at an average of 4,130 pg/ml (Table 1, *n* = 6 mice). A primary highly potent metabolite of tamoxifen is 4-OH-tamoxifen (Robertson et al., 1982); it was present in plasma at 415 pg/ml, about 10% of the tamoxifen level across the samples. In the cerebral cortex of tamoxifen-treated mice, we measured 92 pg/mg tamoxifen and 9.3 pg/mg of 4-OH-tamoxifen (Table 1); 4-OH tamoxifen was 12% of the tamoxifen level. Comparing the concentration of the compounds in the brain to the plasma, we found that the level of tamoxifen and 4-OH-tamoxifen in the brain was over twice that in the plasma. Thus, the administered tamoxifen crosses readily into the CNS of the mouse and its metabolite is present at a constant percentage in the brain and plasma.

**TABLE 1:** Tamoxifen and 4-OH-tamoxifen measures.

Tissue	Tamoxifen	4 OH-tamoxifen	OH-tam/tam
Brain	92 (65) pg/mg	9.3 (1.0) pg/mg	0.12 (0.4)
Plasma	4,130 (1,230) pg/ml	415 (117) pg/ml	0.10 (0.01)
Brain/plasma ratio^1^	2.16 (1.02)	2.35 (0.54)	

Mean (standard deviation), *n* = 6 mice/group. ^1^Ratio of brain to plasma measures based on estimates of a volume of 400 mm^3^ per brain (Kovacević et al., 2005) and 100 μg protein per mg brain wet weight (Ericsson et al., 2007).

### 3.3. Effects of tamoxifen on mouse behavior

For behavioral analyses, two additional cohorts of tamoxifen and vehicle control mice were tested, totaling 16 mice in each of the treatment groups. At the beginning of the behavioral experiments (with mice at 27 weeks of age), the tamoxifen-treated and vehicle control groups were similar in weight. No difference was observed in the weight changes experienced between the groups for the duration of the study (Figure 1). After this chronic exposure, the mice underwent a series of behavioral assessments.

**FIGURE 2:**
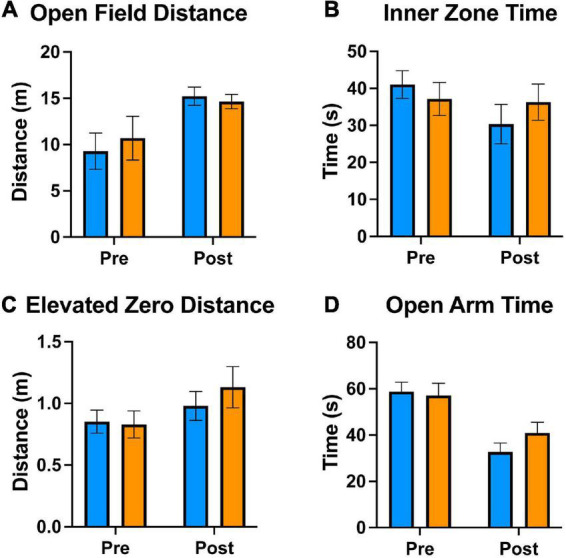
Tamoxifen treatment does not alter locomotive and anxiety behaviors. **(A–D)** Results of Open Field and Elevated Zero behavioral assessments; “pre-treatment” time point 1 week prior to tamoxifen or control exposure, “post-treatment” 2 days following completion of treatment **(A–D)**. **(A)** Distance traveled in a 5 min Open Field exploration task. **(B)** Time spent in the Inner Zone of the Open Field. **(C)** Distance traveled in a 5 min Elevated Zero exploration task. **(D)** Time spent in the Open Zone of the Elevated Zero apparatus. *N* = 16 mice per group; mean ± standard error.

We used the open field and elevated zero mazes to test the effects of tamoxifen on mouse exploration and anxiety. Prior to treatments, the two sets of mice traveled similar distances (Figure 2A) and spent similar amounts of time in the exposed center of the open field apparatus (Figure 2B). Similarly, in the Elevated Zero apparatus, they traveled similar distances (Figure 2C) and spent similar amounts of time in the exposed arms (Figure 2D). After exposure to tamoxifen or vehicle control, both groups of mice again traveled similar distances (Figures 2A, C) and spent similar amounts of time in exposed regions of the Open Field and Elevated Zero mazes (Figures 2B, D). The distance traveled was increased in all groups post-treatment. Thus, tamoxifen exposure did not affect these measures of exploration or anxiety.

**FIGURE 3:**
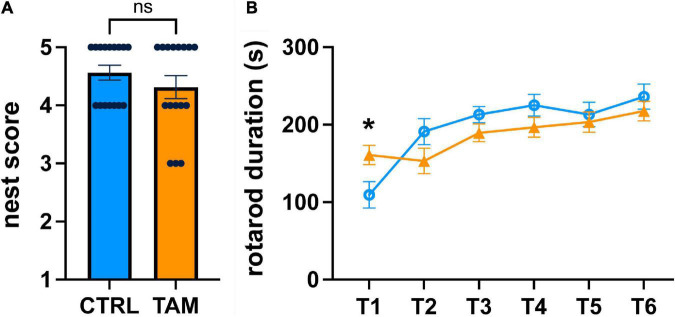
Tamoxifen effects on general health or motor skill learning and coordination. Blue lines and bars show results from control (CTRL) mice; orange lines and bars show tamoxifen-treated mice (TAM). **(A)** Scoring of nests built by single-housed mice overnight based on comparison to well-defined figures ranked from 1 (no nesting) to 5 (full nest). **(B)** Performance on standard motor learning task measured as latency to fall off the accelerating rod from trial 1 (T1) to trial 6 (T6). *N* = 16 mice per group; mean ± standard error. For a comparison of the initial ability to perform the rotorod task, a *t*-test was conducted individually for T1 (*p < 0.02).

For analysis of general health, we used a nest building task (Figure 3A). Mice were given cotton squares, and their ability to construct nests overnight was evaluated. All mice used the material to construct nests to some degree, scored on a scale of 1 (little to no nest) to 5 (complete nest developed). The nests of the control group were scored between 4 and 5, while those of the treatment group were scored between 3 and 5 (Figure 3A). This difference was not statistically significant, indicating that mice in both groups were generally healthy and not experiencing physical discomfort.

We looked at motor learning using the rotorod task (Figure 3B). Mice were tested for their ability to maintain balance on a spinning rod that accelerated over time. During the first rotarod trial, the mice in the tamoxifen-treatment group spent 47% more time on the accelerating rod than those in the control group (*p* < 0.02) (Figure 3B). The time spent on the rotating rod increased over the subsequent five trials, indicating similar positive motor coordination and motor skill learning. There was no statistically significant difference between the two groups during any of these trials, indicating that tamoxifen did not affect motor learning.

**FIGURE 4:**
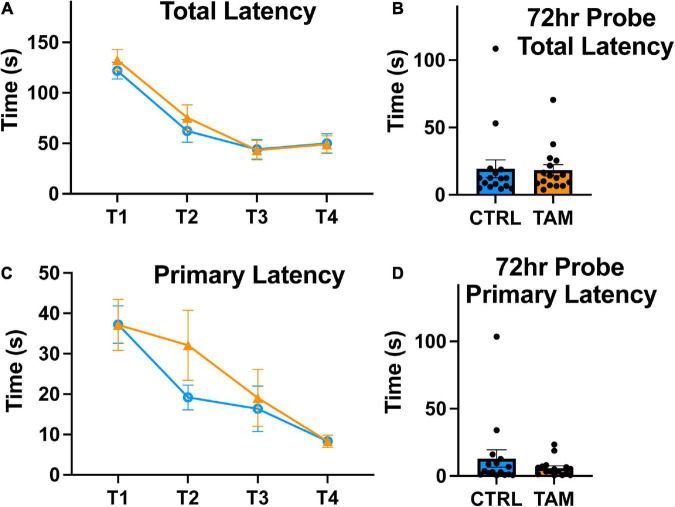
Tamoxifen treatment does not impair spatial learning. Blue lines and bars show results from control mice (CTRL); orange lines and bars show tamoxifen-treated mice (TAM). **(A)** Average latency to full entry into the escape hole of the four trials completed by each mouse across 4 training days (T1-T4). **(B)** Time in seconds to escape from Barnes Maze on probe trial conducted 72 h following the T4. **(C)** Average latency to first approach to escape hole of the four trials completed by each mouse from T1-T4. **(D)** Time in seconds until the first approach to escape hole on probe trial conducted 72 h following the final training trial. *N* = 16 mice per group; mean ± standard error.

We used a Barnes maze to evaluate hippocampus-related spatial learning and memory (Figure 4). Over the first three training days, all mice exhibited reduced time to escape into the target hole, indicative of spatial learning and motivation to escape the exposed maze (Figure 4A). There were no statistically significant differences between the two groups in this total latency. In the probe trial three days later, the mice in both groups escaped the maze in similar times compared to training day 4, indicative of retained memory (Figure 4B). We also assessed primary latency, defined as the time for the mouse to make a first nose poke into the escape hole. Again, the two groups of mice showed clear reductions over training days in the time to find the target hole (Figure 4C). There were no significant differences in the time it took between the tamoxifen-treated and control mice. In the three-day probe trial, mice again demonstrated the reduced time to identify the target hole and there were no significant differences in that time between groups (Figure 4D).

**FIGURE 5:**
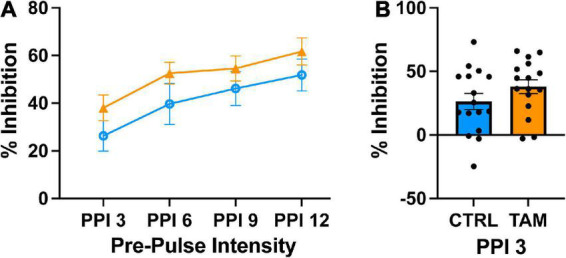
Tamoxifen treatment does not alter pre-pulse inhibition (PPI). Blue lines and bars show results from control mice (CTRL); orange lines and bars show tamoxifen-treated mice (TAM). **(A)** Percent inhibition of startle response to a loud tone following a pre-pulse of 3, 6, 9, or 12 decibels (dB) above background noise (PP3–PP12) compared to startle with no pre-pulse. **(B)** Percent inhibition of startle response to a short tone following the pre-pulse 3 decibels above background noise compared to startle with no pre-pulse 2 weeks post-treatment. *N* = 16 mice per group; mean ± standard error.

We tested sensorimotor gating in the mice using a pre-pulse inhibition task (Figure 5). In an enclosed apparatus, mice are startled by a pulse of loud sound, but pre-pulse stimuli of various intensities inhibit the magnitude of the startle. As expected, the louder pre-pulse stimuli caused more startle inhibition (Figure 5A). The pre-pulse of 3 dB above background caused about 30% startle inhibition across groups (Figure 5B), without significant differences between them. At 3 dB, the tamoxifen treated mice experienced higher startle inhibition than the control group across all pre-pulse stimuli, although this difference was not statistically significant. Similar results were observed at the other pre-pulse intensities.

**FIGURE 6:**
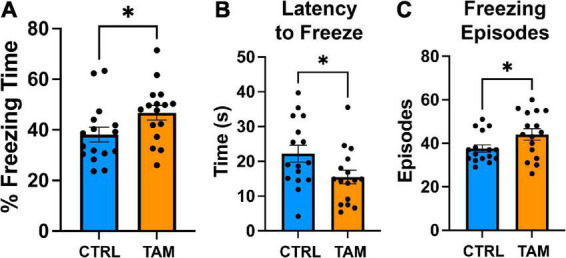
Tamoxifen increases contextual fear response. Blue bars show results from control mice (CTRL); orange bars show tamoxifen-treated mice (TAM). **(A)** Percent time spent freezing in the conditioned environment 48 h post-training without shock. **(B)** Latency to freeze in the conditioned environment 48 h post-training without shock. **(C)** Number of freezing episodes in the conditioned environment 48 h post-training without shock. *N* = 16 mice per group; mean ± standard error. *p < 0.05, unpaired *t*-test.

Finally, a contextual fear conditioning task was used to assess amygdala-related learning and memory (Figure 6). Prior to the foot shock, mice froze 17% of the time (control mice, 16.9%; tamoxifen-treated mice, 16.5%). Two days after initial foot shocks, mice demonstrated freezing for about 40% of the time in the conditioned apparatus. Mice in the tamoxifen treated group froze a significantly longer period of time than those in the control group (Figure 6A; *p* = 0.04). This increase in time was accompanied by more freezing episodes (Figure 6C, *p* < 0.05) and a shorter latency to freeze (Figure 6B; *p* = 0.04).

### 3.4. Next-generation RNA sequencing and transcriptomic profile

**FIGURE 7:**
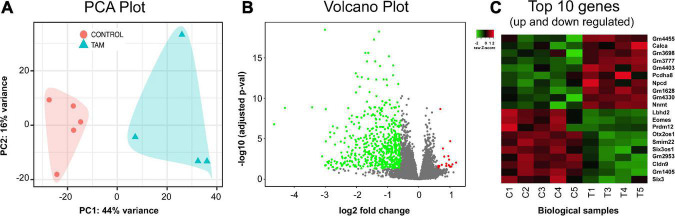
RNA-seq analysis and transcriptome profile. Hippocampus mRNA differential expressional levels between control (*n* = 5) and tamoxifen-treated (TAM) (*n* = 4) mice. **(A)** Principal component analysis (PCA) of all RNA-seq hippocampal samples. Control group (red) and tamoxifen group (blue) are clustering distinctly. **(B)** Volcano plot analysis summarizing differentially expressed genes (DEGs) of tamoxifen-treated group vs. control group of animals. A total of 669 genes were identified as significant DEGs, out of which 647 (green) genes were found to be down-regulated and 22 (red) genes were up-regulated in TAM treated animals compared to controls. **(C)** Heat plot summarizing top 20 genes (10 up-regulated and 10 down-regulated) based on lowest adjusted *p*-value (FDR = 0.05, fold change cut-off = 1.5). Details of these genes are also listed in Table 2.

For comprehensive characterization of the effects of chronic tamoxifen-treatment on brain mRNA, we analyzed hippocampus transcriptomic profiles of 5 control and 5 tamoxifen-treated mice. We chose the hippocampus for its importance in the consolidation of new memories. We sequenced polyA-selected mRNA from hippocampus and achieved total of ∼420 million paired-end reads, with about 40 million reads per sample and an average unique fragment mappability of ∼93%. One sample from a tamoxifen-treated mouse was excluded after hierarchical clustering using Pearson correlation matrix analysis. Control animals and tamoxifen-treated animals were distinctly separated when analyzed using principal component analysis (PCA). The first principal component explained a large amount, 44%, of the variance of the data (Figure 7A).

A total of 669 genes were significantly differentially expressed between control and tamoxifen-treated groups (Figure 7B); 647 genes were down-regulated and 22 genes were up-regulated in the tamoxifen-treated group compared to the control group. The top 10 significantly up-regulated and down-regulated genes based on fold-changes were plotted as heat-map (Figure 7C), and are listed in Table 2. We analyzed the pathway enrichment using gene-set enrichment analysis for biological pathways (pre-ranked fgsea package), and identified the top 15 significantly enriched pathways (Table 3). The top down-regulated pathways were related to cilium movement as well as microtubule bundling; these pathways contain many genes related to microtubule assembly, regulation, and transport motors (examples of genes identified: *DNAI3, ODAD4, CFAP43, CFAP 206, ODAD2, FOXJ1, HYDIN*). The most significant pathway that was up-regulated was for genes involved in the negative regulation of dendritic spine morphogenesis (genes identified: *PREN, UBE3A, NGEF, EFNA1, NLGN3, DNM3, DTNBP1, NLGN1*). We also compared our list of tamoxifen-regulated genes to curated hallmark dataset of early and late estrogen response genes (Liberzon et al., 2015). There were 20 overlapping genes: (*HSPB8, CISH, SEMA3B, CA12, ALDH3B1, MYOF, TJP3, MYB, KRT8, TSKU, RHOD, ZNF185, CD9, ACOX2, ST6GALNAC2, LLGL2, IL17RB, CLIC3, ST14, PRLR*). All were down-regulated, consistent with the role of tamoxifen as an estrogen receptor antagonist. The complete list of all differentially expressed genes is provided in the Supplementary File 1.

**TABLE 2:** Top 20 differentially expressed genes based on fold changes (10 up-regulated, 10 down-regulated by tamoxifen).

	Symbol	Ensembl ID	Adjusted *p*-value	log2 fold change	Chr	Type
Up	Gm44559	ENSMUSG00000108943	9.07E-03	1.196	7q	TEC^#^
	Calca	ENSMUSG00000030669	1.50E-02	1.079	7q	protein_coding
	Gm36989	ENSMUSG00000102496	2.74E-02	1.045	18q	TEC
	Gm37773	ENSMUSG00000104033	2.07E-05	1.007	3q	TEC
	Gm44033	ENSMUSG00000108154	2.91E-02	1.003	6q	TEC
	Pcdha8	ENSMUSG00000103800	2.21E-02	0.979	18q	protein_coding
	Npcd	ENSMUSG00000089837	3.88E-03	0.969	15q	protein_coding
	Gm16287	ENSMUSG00000073739	1.84E-02	0.959	4q	lncRNA
	Gm43300	ENSMUSG00000105572	1.41E-03	0.948	3q	TEC
	Nnmt	ENSMUSG00000032271	1.21E-02	0.884	9q	protein_coding
Down	Lbhd2	ENSMUSG00000087075	1.71E-07	-4.643	12q	protein_coding
	Eomes	ENSMUSG00000032446	1.57E-09	-4.297	9q	protein_coding
	Prdm12	ENSMUSG00000079466	4.69E-03	-3.485	2q	protein_coding
	Otx2os1	ENSMUSG00000098682	1.39E-09	-3.424	14q	lncRNA
	Smim22	ENSMUSG00000096215	3.07E-05	-3.395	16q	protein_coding
	Six3os1	ENSMUSG00000093460	1.58E-03	-3.104	17q	lncRNA
	Gm29538	ENSMUSG00000099553	1.58E-04	-3.080	1q	lncRNA
	Cldn9	ENSMUSG00000066720	6.32E-04	-3.038	17q	protein_coding
	Gm14051	ENSMUSG00000086756	6.05E-05	-3.026	2q	lncRNA
	Six3	ENSMUSG00000038805	2.07E-02	-3.011	17q	protein_coding

^#^TEC (To be experimentally confirmed): Regions with EST clusters that have polyA features that could indicate the presence of protein coding genes.

**TABLE 3:** Gene set enrichment analysis (GSEA)–biological pathways.

	GSEA analysis: Tamoxifen vs. control	NES	Genes	Q value
Down	Axoneme assembly	-2.23	72	0.0016
	Cilium movement	-2.21	133	0.0016
	Microtubule bundle formation	-2.17	105	0.0016
	Motile cilium assembly	-2.11	44	0.0016
	Cilium or flagellum-dependent cell motility	-2.09	95	0.0016
	Cilium-dependent cell motility	-2.09	95	0.0016
	Cilium movement involved in cell motility	-2.09	87	0.0016
	Extracellular transport	-2.06	34	0.0016
	Epithelial cilium movement involved in extracellular fluid movement	-2.05	31	0.0016
Up	Negative regulation of dendritic spine morphogenesis	2.16	8	0.0028
	Inhibitory synapse assembly	2.09	15	0.01
	Protein localization to post-synaptic specialization membrane	2.07	29	0.014
	Neurotransmitter receptor localization to post-synaptic specialization membrane	2.07	29	0.014
	Protein localization to synapse	2.07	83	0.01
	Synaptic membrane adhesion	2.05	31	0.016

NES, normalized enrichment score.

## 4. Discussion

Our study is among the first to examine the behavioral and neuronal effects of tamoxifen treatment on mice in the framework of its role in treating breast cancer. We used a mouse model of long-duration tamoxifen treatment to approximate the exposure of breast cancer patients. We found that there were no effects on tamoxifen on behavioral assays related to general health, exploration, motor learning, spatial learning and memory, and sensorimotor gating. We did observe that tamoxifen significantly increased responses to a fear-inducing stimulus; measures of baseline anxiety-related behaviors were unaffected. In addition, we used RNASeq analysis to demonstrate that tamoxifen-treated mice had pronounced reductions in expression of genes related to the normal regulation of cilium microtubules. These data reveal that chronic tamoxifen exposure has demonstrable effects on brain function in mice.

We were able to reproduce the earlier work demonstrating that after tamoxifen exposure, tamoxifen and its active metabolite 4-OH-tamoxifen are present in the brain (Lien et al., 1991). Tamoxifen and 4-OH-tamoxifen were measured one day after the final drug administration in a cohort of mice that were not behaviorally tested. This protocol allowed us to separate the long-term effects of tamoxifen on behavior from the short-terms effects of behavior on gene expression. Across the samples that we analyzed, the levels of tamoxifen and 4-OH-tamoxifen in the blood correlated well with the levels in the brain, making it possible to infer relative brain tamoxifen levels from the plasma levels. In mice, tamoxifen is routinely used in transgenic models to regulate induction of transgenes through estrogen receptor binding, further emphasizing that tamoxifen readily enters the brain (Donocoff et al., 2020). The brain effects of tamoxifen observed here could be mediated through many molecules, including estrogen receptors α and β, and G protein-coupled estrogen receptors (Novick et al., 2020).

Tamoxifen is metabolized differently in humans, mice, and rats (Robinson et al., 1991), leading to different levels of tamoxifen, 4-OH-tamoxifen, and another major tamoxifen metabolite, N-desmethyltamoxifen (Robinson et al., 1991). The levels of tamoxifen achieved in the plasma of the mice treated here are over ten-fold lower than what is observed in human serum, based on oral dosing in humans of approximately 20 mg per day (Kisanga et al., 2004; Furlanut et al., 2007). The ratio of 4-OH-tamoxifen to tamoxifen in our study was similar to that seen in humans, about 10% (Kisanga et al., 2004; Furlanut et al., 2007). We used a dose of tamoxifen based on a dose common in humans, correcting for the generally higher metabolism of compounds in mice and differences in administration (orally in humans, subcutaneously in this study) (Nair and Jacob, 2016). Similar doses were used in other rodent studies (Chen et al., 2002; Perry et al., 2005; Scalzo et al., 2021; Sahafi et al., 2022), although higher doses of tamoxifen were used in some studies (Li et al., 2009; Walker et al., 2011; Pandey et al., 2016; Lee et al., 2020) and lower doses used in others (Zhang et al., 2021). Although the current study is important for understanding the effects of tamoxifen through its most avid binding targets, our concurrent measures of plasma and brain tamoxifen demonstrate that higher doses are needed to model the more general effects of tamoxifen.

Tamoxifen has been associated with hypometabolism in the frontal cortex and impaired memory (Eberling et al., 2004). Some clinical studies have failed to find an effect of endocrine therapies on cognitive function in breast cancer survivors, although concerns have been raised about the sensitivity of tests to subtle problems (Hermelink et al., 2008; Breckenridge et al., 2012; Dyk et al., 2019). Our general finding that tamoxifen treatment had limited behavioral effects in mice echoes the negative findings of other pre-clinical studies (Novick et al., 2020). There are a few considerations related to the use of mouse models to investigate the cognitive effects of tamoxifen. Mouse models allow for reproducibility of treatment exposure and control for potential effects of the cancer itself on cognition (Novick et al., 2020). Conversely, women with breast cancer are recommended to be treated with tamoxifen for 5–10 years and mouse models must be done over shorter times. Furthermore, language cannot be tested in mice, and it may be one of the more vulnerable cognitive domains to the effects of tamoxifen (Jebahi et al., 2021). While our findings are reassuring about there being no dramatic cognitive effects of tamoxifen on memory function, it remains that some women do report subjective cognitive changes on tamoxifen. While it is possible that subjective cognitive changes are clustered with other symptoms such as anxiety, ongoing clinical and pre-clinical studies are needed to fully interrogate more subtle effects.

We found a significant effect of tamoxifen treatment on a fear conditioning task, such that tamoxifen treated mice spent more time freezing in the context where the experimental shock occurred. Thus, tamoxifen did not decrease the ability to learn to associate a context with a fear stimulus; it either heightened the fear experienced or decreased the extinguishing of the fear that occurs over time (Xu et al., 2019). Given the behavioral assay variability, the significant effects of tamoxifen on fear levels should be re-tested in future studies, along with the any effects on the extinguishing of fear responses (Milad et al., 2009). There is greater anxiety-like behavior in tamoxifen-treated mice during the elevated maze task (Li et al., 2020), and increased anxiety behaviors in rhesus macaques during tamoxifen treatment (Mook et al., 2005). We did not observe any anxiety like behaviors in unchallenged tamoxifen-treated mice, suggesting that the effects may become more noticeable under stressful conditions. From a clinical standpoint, this finding is important to pursue and further investigate. Women with breast cancer experience substantially higher levels of anxiety than the general population, with prevalence rates around 40% (Hashemi et al., 2020). Elevated symptoms of trauma are also observed in women with breast cancer, including symptoms of post-traumatic stress disorder stemming from diagnosis and ongoing monitoring (Arnaboldi et al., 2017). It will therefore be important to better understand if tamoxifen interferes with stress responses for these patients, especially in the context of breast cancer-related stressors (Parikh et al., 2015).

Animal and cell models have identified several effects of estrogen on neuron function; since tamoxifen is a selective estrogen receptor modulator, it can affect similar processes. Estrogen promotes growth of neuronal processes and facilitates neuroplasticity (Prange-Kiel and Rune, 2006; McEwen et al., 2012). Increased estrogen levels during an animal’s estrous cycle increases in hippocampal spine density (Kato et al., 2013) and affects processes of long-term potentiation (Warren et al., 1995; Good et al., 1999). In the hypothalamus, tamoxifen treatment resulted in down-regulation of pathways of neuropeptide signaling through effects on the estrogen receptor Esr1 (Zhang et al., 2021). *In vitro*, estrogen facilitates mossy fiber sprouting and spine density in hippocampal neurons (Murphy and Segal, 1996; Teter et al., 1999). Studies that have investigated tamoxifen treatment on neurites have led to mixed results; tamoxifen has been found to both facilitate synaptic density and down-regulate it (Murphy and Segal, 1996; Silva et al., 2000). In humans, breast cancer survivors undergoing tamoxifen and adjuvant treatments treatment showed reduced hippocampal function connectivity (Chen et al., 2017), suggesting that the hippocampus is a vulnerable target during tamoxifen treatment.

Here we found that tamoxifen had the most significant effects on down-regulating pathways related to microtubules that are important to cilium function and to cell cycle regulation, which are dysregulated in neurodegenerative processes (Youn and Han, 2018; Ma et al., 2022). The pathway “negative regulation of dendritic spine morphogenesis” was the most significantly up-regulated, also suggesting effects of tamoxifen on normal regulation of axon targets at synapses. The importance of these pathways is supported by other studies on the effects of altering estrogen receptor genes in the brain. Analysis of the effects of tamoxifen on prenatal and developing mice found impairments in neurogenesis in neurodevelopment and into adulthood (Lee et al., 2020). Estrogen treatment of rodents over one week to one month showed diverse effects on cortex and hippocampal gene expression (Humphreys et al., 2014; Sárvári et al., 2015), including transcripts related to growth factors and development that were also observed in our study (*IGFBPL1, EOMES, OTX2, TNS4, CDKN1*). The hippocampal mRNA effects found in our current study occur within the limited levels of plasma tamoxifen achieved, supporting the interpretation that they are among the most sensitive responses to tamoxifen. These pathways affected by tamoxifen remain speculative, but strongly implicated are the processes of hippocampal neurogenesis (Mahmoud et al., 2016) and synaptic plasticity (Sheppard et al., 2019), both of which are altered in normal aging and Alzheimer’s disease (Chen et al., 2023).

Although tamoxifen has been studied in animal models in a variety of other contexts, they have rarely been studied in models oriented toward breast cancer treatment with behavior and brain tissue-derived markers as the primary outcomes, a strength of our study. We estimated what dose might approximate human exposures based on existing reports but found that future studies would need use a higher dose to model concentrations similar to those seen in humans. Even at this dose, however, we observed noteworthy effects of tamoxifen on both behavior and gene expression in hippocampal neurons. There are several important follow-up studies to these findings including determining the specific effects of tamoxifen on brain structure and extending this inquiry into other anti-estrogen breast cancer treatments (e.g., aromatase inhibitors). Given the frequency and extent of treatment in women with breast cancer, fully investigating the behavioral and neuronal effects of anti-estrogen treatments like tamoxifen remains a clinical imperative.

## Data availability statement

The data that support the findings of this study are available from the corresponding author upon reasonable request. All next-generation sequencing raw data for RNA-seq experiments are available from SRA (https://www.ncbi.nlm.nih.gov/sra/PRJNA911742), data accession # PRJNA911742.

## Ethics statement

The animal study was reviewed and approved by Georgetown University Medical Center Institutional Animal Care and Use Committee.

## Author contributions

GR and KV designed the experiments, interpreted the results, and wrote the manuscript. EG conducted the behavioral experiments. HP conducted the transcriptomic analysis and interpretations. JS, CN, and MB conducted the mouse experiments. JM developed the original idea and designed the experimental approach. All authors edited the manuscript.
